# A Tale of Two Capitals: How Task-Oriented and Guanxi-Oriented Psychological Capitals Lead to a Sustainable Workforce in Rural China

**DOI:** 10.3389/fpsyg.2021.732445

**Published:** 2021-08-31

**Authors:** Chunyan Xu, Dawei Wei, Jintao Liu, Jiaxian Zhou

**Affiliations:** ^1^Department of Psychology, School of Education, Lanzhou City University, Lanzhou, China; ^2^Department of Educational Psychology, Center for Educational Neuroscience, Faculty of Education, East China Normal University, Shanghai, China; ^3^Department of Applied Linguistics, School of Humanities and Social Sciences, Xi’an Jiaotong-Liverpool University, Suzhou, China; ^4^Language and Cognition Lab, Department of English, Lanzhou Jiaotong University, Lanzhou, China

**Keywords:** psychological capital, task-oriented psychological capital, guanxi-oriented psychological capital, organizational commitment, vocational identity, job satisfaction, gongwuyuan cadres

## Abstract

Psychological capital (PsyCap) is documented to be positive in influencing employees’ behavior. However, little attention has been paid to its role in maintaining a sustainable workforce in underprivileged rural areas. Also less known is the complex relations between PsyCap and other consequence variables. Moreover, previous studies in this field did not adequately address the cross-cultural applications of positive resources, though many facets of PsyCap are culture related. To address the gaps, the current study explored the complex relationships linking PsyCap and organizational commitment in a sample of public civil servants (gongwuyuan cadres, *n*=583) at the township level in the rural areas of northwestern China. Two types of PsyCap, task-oriented PsyCap, which is similar to the PsyCap in the west, and guanxi-oriented PsyCap, which is unique in the Chinese culture, were measured. Task-oriented PsyCap is composed of enterprise-diligence, resiliency-perseverance, optimism-hope, and confidence-courage. Guanxi-oriented PsyCap is composed of toleration-forgiveness, modesty-prudence, thanksgiving-dedication, and respect-courtesy. AMOS 23.0 software was used to establish structural equation models. The results show that both types of PsyCap were positive predictors of organizational commitment. Vocational identity and job satisfaction mediated the relation between task-oriented PsyCap/guanxi-oriented PsyCap and organizational commitment. The chained relationship from the two types of PsyCap to vocational identity, job satisfaction, and organizational commitment was also significant. These results and their implications for workforce stability are discussed.

## Introduction

Over the last several decades, increasing attention has been shifted to the organizational dynamics in the developing economies around the world. In China, with the central government’s Targeted Poverty Alleviation program in 2013, the national efforts in reducing poverty have since pivoted to the rural areas (Guo et al., 2019). As such, the grassroots public service sector has expanded steadily, with increasing workloads placed on the local civil servants in the anti-poverty campaigns (Lin, 2014) as well as general public affairs administration. Their work is characterized as a stressful and intensive job with relatively low income and heavy work investment (Snir and Harpaz, 2009) typically driven by the compulsion to work. As a cohort of workers within close contact with the general public in the underdeveloped rural areas, they face challenges of managing public affairs and utilities. Studies show among the grassroots public sector employees, their highly stressful jobs have caused an increasing turnover (Shi and Zhao, 2014) and have had negative impacts on their physical and mental health (Yu and Chen, 2020). On the other hand, these grassroots gongwuyuan cadres are a part of “post 1980s/1990s generations.” Most of them have grown up under the “Reform and Opening-up” policy in 1978 and as a result, have experienced major socioeconomic and cultural changes. Similar to Generation Y in the west, this new generation of employees in China is characterized as being more autonomous, self-centered with diversified career ideals and paying more attention to the work-life balance than their predecessors (Zhu et al., 2015). Previous studies found that they tend to resist traditional hierarchy, distrust workplace institutions, and challenge the workplace through rapid turnover (Cogin, 2012; Chung and Fitzsimons, 2013; Chen et al., 2016). Given the increasingly aging society with a low birth rate in China (Zhu et al., 2019), the current employee workforce, especially the new generations post the 1980s, has been important for employers to reflect on their management models and to boost their employees’ motivation and commitment to their organizations.

While previous research has documented that the employees’ behavior and motivation in their positions are influenced by the resources of positive psychology, such as psychological capital (PsyCap), few studies have attempted to uncover the potential benefits of PsyCap in maintaining a sustainable public workforce and improving stressed staff performance especially in the underprivileged areas (Shang Guan et al., 2017). Though previous studies have explored how PsyCap may impact some consequence variables, such as work performance (Luthans et al., 2007a; Rabenu et al., 2017; Nolzen, 2018), more efforts are needed to explore the path leading to a steady workforce as a whole and to untangle the complexities between relevant variables concerning PsyCap. The current study aims to fill these gaps and examines the complex relationship between PsyCap and other consequence factors of job satisfaction, vocational identity, and organizational commitment in the public sector workforce in the less developed rural townships^1^ of northwestern China.

## Theoretical Foundations and Hypotheses

The Conservation of Resources (COR) theory proposes that individuals who possess positive resources “might be more capable of selecting, altering, and implementing their other resources to meet stressful demands” (Hobfoll, 1989, 2002). The stressors are likely to dampen employees’ motivational behaviors, thwart work performance, and discourage personal growth (Lepine et al., 2005). Previous studies have shown the relevance of COR theory as a theoretical foundation for the importance of individual’s positive psychological resources to cope with stressors, such as negative organizational politics and economic hardship (Hobfoll et al., 2018). In the underprivileged environment, the grassroots gongwuyuan cadres in the current study must leverage their positive emotions as well as other social supports to carry out their roles and to meet the expectations from their organizations and communities. As a motivational theory, COR theory is basic and evolutionary in explaining much of how human beings conserve resources to survive. Therefore, it is appropriate to rely on COR theory as a starting point to examine how positive psychological resources of the cadres might influence job-related outcomes. In line with COR theory, broaden-and-build theory (Fredrickson, 2001) and in particular positive psychology theory (Seligman and Csikszentmihalyi, 2000) are also relevant in building up our hypotheses. The broaden-and-build theory proposes that people’s positive emotions broaden their “thought–action repertoires,” resulting in actions that are wider than what they typically do. In the social and interpersonal context, this theory predicts that experiences of positive emotions broaden people’s sense of self so that their identification with others are enhanced and consequently the feeling of self-other overlap and “oneness” is gradually achieved (Waugh and Fredrickson, 2006). With the above theories we argue that in the case of the current study, the grassroots cadres with high levels of PsyCap would possess capacity to derive pathways to be successful and perseverance in the face of adversities (Stajkovic and Luthans, 1998). They tend to be more satisfied with their job and gradually develop their identification with and commitment to the organization they work for.

In the late 1990s, positive psychology gained momentum and saw its influence across various fields, such as business and management. Instead of traditional psychology with its focus on weakness, pathology, and mental problems, positive psychology is defined as “the scientific study of what goes right in life” (Peterson, 2006). It explores more positive view of human mind, such as strengths, virtues, and characters to enable a productive and flourishing functioning of individuals, groups, and organizations. Under the influence of positive psychology as a theoretical rationale, Luthans initiated a positive organizational behavioral (POB) movement (Luthans, 2002). Originated in economics studies, PsyCap was initially an important form of capital in impacting wages (Goldsmith et al., 1997). Luthans and his colleagues later singled out PsyCap as fundamentally “who you are” (Luthans and Avolio, 2003) from “what you know” [or, human capital, (O'leary et al., 2002)] and “who you know”(or, social capital, Adler and Kwon, 2002). To be specific, PsyCap is defined as a core psychological factor of positivity in general, and POB criteria meeting states in particular, that go beyond human and social capital to gain a competitive advantage through investment/development of “who you are.” To measure PsyCap, various dimensions have been put forward. Optimism, hope, and resiliency and self-confidence/self-efficacy are the main components of PsyCap (Larson and Luthans, 2006). More aspects, such as creativity, wisdom, humor, and gratitude, are also regarded as factors in building a PsyCap composite (Luthans et al., 2007b). Since then, the last decade has seen a rapid increase in the amount of research on PsyCap, including its measurement and effects in various areas, such as in sports (Kim et al., 2019), education (Carmona-Halty et al., 2021; Siu et al., 2021), and management (Nolzen, 2018).

Meanwhile, it is also cautioned that cross-cultural differences need to be considered in developing the construct, as the subordinate concepts, such as self-efficacy, optimism, and resiliency, are subject to individual differences and external contexts (Luthans et al., 2007b; Newman et al., 2014). In fact, some studies revealed it is necessary to take cultural context into account. For example, some items of the Resilience Scale (Wagnild and Young, 1993) did not apply to a sample of 450 Soviet immigrants formerly living in the collectivist culture (Aroian et al., 1997). In a study targeting professional managers working in the oil and gas industry in Saudi Arabia, Idris and Manganaro (2017) did not find an association between PsyCap and organizational commitment, regardless of covariates. They speculated that a cultural difference from the west might be one of the reasons leading to the null correlation as the participants tended to select middle responses in organizational commitment and job satisfaction measurements, resulting in significantly different standard derivations of the variables. The other reason might lie in the language use, also a crucial part of a culture, in their investigation. The English language, rather than the native Arabic of the participants, was used in the survey questions.

Luthans and colleagues also argue for the difference in the resource sets to be obtained over the life spans of people in different cultural contexts (Luthans et al., 2007b). In contrast to western culture, Chinese culture lays much more emphasis on social interpersonal connections, or “guanxi” in Chinese. As Fei (1992) pointed out, different from the American society featuring voluntary associations on the basis of universalistic principles, the social fabrics in China are organized by guanxi circles. Not surprisingly, Guanxi is regarded as essential in gaining access and approval in almost every realm of life including business and politics (Lee and Dawes, 2005). At both levels of organizations and individuals, conflicts in guanxi with others could cause ineffective communications and even less successful work performance and career.

Based on the POB meeting criteria and rooted in the Chinese culture, the local PsyCap Scale is tested to include two high-order constructs of task-oriented PsyCap and guanxi-oriented PsyCap (Ke et al., 2009). The task-oriented PsyCap has four sets of factors of enterprise and diligence, resiliency and perseverance, optimism and hope, and confidence and courage. Guanxi-oriented PsyCap is composed of toleration and forgiveness, modesty and prudence, thanksgiving and dedication, and respect and courtesy. To some extent, the task-oriented PsyCap is more inclusive than its counterpart in the west (Larson and Luthans, 2006) but the two scales are “basically similar” with a high correlation coefficient of 0.70 (Ke et al., 2009, p 882). The guanxi-oriented PsyCap is found to be unique in the Chinese context with its strong connection with traditional Chinese values. Since the first report by Ke et al. (2009), other studies replicated the guanxi-oriented PsyCap in Chinese primary and middle school teachers (Wu et al., 2012), doctors (Sun et al., 2014), employees in a company and a local government in Beijing (Ke and Sun, 2014), and those working in the aviation industry (Wang, 2020). These studies provide evidence for the properties of guanxi-oriented PsyCap. According to Ke et al. (2009), guanxi-oriented PsyCap as well as task-oriented PsyCap met the POB criteria of “being measurable, open to development, and can be managed for more effective work performance” (Luthans, 2002). The construct of guanxi-oriented PsyCap was set up with the following two steps, which showed that the PsyCap is measurable. First, to collect measurement items of the local PsyCap scale, various methods were used including in-depth interview, literature review, successful people’s biography material collection, and unstructured questionnaire survey. Then, item analysis, reliability analysis, factor analysis, correlation analysis, and regression analysis were used to test the reliability and validity of the scale. The four sets of characteristics of guanxi-oriented PsyCap are widely regarded as critical moral characters in traditional Chinese philosophy. They are largely advocated and expected in social communications in modern China. They are cultivated not only by people themselves (Ivanhoe, 2000; Ke et al., 2009) but also promoted in education (Yang and Liu, 2020) and managed in business settings (Han et al., 2012; Tsui et al., 2017). Also in Ke et al. (2009), high-performance employees were invited in an open questionnaire survey for their state of mind and later interviewed. This ensured that the characteristics of PsyCap contributed to work performance. In sum, guanxi-oriented PsyCap abides by the POB meeting criterion and can be measured, improved, and managed for effective performance. The local PsyCap, covering both task- and guanxi-oriented constructs, can explain larger variances of work performance than the PsyCap counterpart in the west (Ke and Sun, 2014). To provide a complete picture of the positive psychological sources specific to the concerned grassroots servants in the Chinese cultural setting, the local PsyCap and its measurement composed of task and guanxi facets are used in the current study.

Two lines of research in relation to PsyCap can be found in recent years. One is the study of the antecedents of PsyCap, i.e., the factors that influence PsyCap, such as organizational culture, leadership, and perceived organizational support (Wang et al., 2017). The other is concerned with the outcomes of PsyCap, such as organizational commitment, job satisfaction, and subjective wellbeing (Li, 2018). Organizational commitment is an employee’s psychological attachment to their organization which over a period of time helps maintain membership in an organization (Meyer et al., 2002). It is linked to the degree to which the individual employee identifies with and makes efforts to achieve the common organizational goals (Allen and Meyer, 1993). Various conception frames of organizational commitment have been formulated. For example, Meyer and Allen suggested three components of affective, continuance, and normative commitment (Allen and Meyer, 1993). Affective commitment focuses on the emotional bond that employees feel toward their organization. Continuance commitment is the awareness of employees of the cost of leaving the organization. Normative commitment is defined as the individual’s attachment with the organization as a result of an obligation on the part of the individual.

In a study exploring the correlation between volunteers’ PsyCap and their commitment to volunteer service, Xu et al. (2020) reported a positive influence of the PsyCap on their organizational commitment. The PsyCap in their study refers to the traditional version covering self-efficacy, optimism, hope, and resilience (Luthans and Youssef, 2007). Still in a Chinese work environment, in a sample characterizing public servants in a local government and employees in a company in Beijing, Ke and Sun (2014) found both task-oriented PsyCap and guanxi-oriented PsyCap are significantly related with organizational commitment, with the latter correlation much stronger than the former one (0.21 vs. 0.47). The gongwuyuan cadres concerned in the current study share the same Chinese culture with those organizational employees’ investigated (Ke and Sun, 2014). With previous studies and in line with COR theory and broaden-and-build theory, it is hypothesized that as follows:

*H1a*: Task-oriented PsyCap will be a significant predictor of organizational commitment of grassroots gongwuyuan cadres.

*H1b*: Guanxi-oriented PsyCap will be a significant predictor of organizational commitment of grassroots gongwuyuan cadres.

It is later found that the positive effect on individuals and their organizations from their PsyCap is indirect, with some mediating or moderating variables functioning between the outcome variables and PsyCap (Culbertson et al., 2010). One of the potential mediating variables is vocational identity. Vocational identity is a crucial factor in the employee’s career development. It plays critical roles in job-related choices, occupational directions, transitions from training/education to workplace, and career prospects by serving as “a strong cognitive and affective foundation for dispositional employability” (Porfeli et al., 2011). According to social identity theory, people have an innate need for categorization and tend to classify themselves into certain groups with membership in a group (Turner and Oakes, 1986). In such social groups, they construct their identity by learning the common language, participating in its social activities, appreciating, and adapting themselves to the culture of the group. In this way, they gain higher self-esteem and cognitive security and enjoy a sense of belonging and develop their personality (Turner and Oakes, 1986). Therefore, the elements of a worker’s social identity may reflect how the membership in a group impacts them intellectually and emotionally and followers in terms of their intergroup behavior. It is reported that a strong identification with a workgroup is correlated with increased employee motivation and organizational trust and commitment (Van Dick et al., 2005). It enhances employee’s job satisfaction (Ullrich et al., 2005) and increases job performance and organizational effectiveness (Chughtai and Buckley, 2010). According to this theory, the gongwuyuan cadres’ identification with their profession in the local government may influence their personal opinions on their jobs and their organizational commitment. Variables, such as individual-related factors (demographic dimensions, self-efficacy, response style, and self-esteem), family-related factors (parenting, family climate), and types of organization, have been found to influence vocational identity. In a study investigating how the western PsyCap related to vocational identity in Chinese nurses (Wang et al., 2013), a sample of 455 nurses across five large top hospitals were surveyed. There existed a significant correlation between PsyCap and vocational identity (*p*<0.01). The dimensions of self-efficacy and optimism in PsyCap were positive in building a healthy vocational identity of the nurses. As for the guanxi-oriented PsyCap, there have been few studies on how it may impact vocational identity. But according to Vuyk et al. (2020), guanxi, which is pervasive between individuals and organizations in China, to some extent affects undergraduates’ career choice and development. They are more likely to exchange vocational experiences in social communication, as it could benefit their future career. So the attributes in guanxi-oriented PsyCap, such as modesty, toleration, and respect, might help maintain good social contexts, which in turn may contribute to their vocational identity. On the other hand, vocational identity guides employees to enact and sustain their behaviors consistent with their self-view and promote their employability (Fugate and Kinicki, 2008). Their dispositional employability was significantly associated with employees’ positive emotions; their affective commitment was related to organizational changes (Fugate and Kinicki, 2008). In a sample of Chinese college teachers, a significant correlation was found between vocational identity and organizational commitment (Sun and Ye, 2015). Tasked with increasing teaching load and research output, the teachers in Chinese higher education institutions tended to complain about their careers to some extent. This has been reflected in their relatively low level of commitment to their colleges as well as their teaching job. Based on the social identity theory and previous investigations, we formulate the following hypothesis:

*H2a*: Vocational identity will act as a mediator between task-oriented PsyCap and organizational commitment.

*H2b*: Vocational identity will act as a mediator between guanxi-oriented PsyCap and organizational commitment.

Apart from vocational identity, another important variable is job satisfaction. As one of the most intensively studied variables in organizational behavior domain, job satisfaction is defined as “a pleasurable or positive emotional state resulting from the appraisal of one’s job or job experiences” (Locke, 1976, p 1304). Job satisfaction can be divided into intrinsic job satisfaction and extrinsic job satisfaction (Cooper-Hakim and Viswesvaran, 2005). Extrinsic satisfaction is the ability of a job to bring value to employees, such as salary and benefits, interpersonal relationships, and the physical conditions of the work environment. Intrinsic job satisfaction is the pleasant emotions and positive attitudes that a job brings to employees, including the sense of achievement, creativity, and opportunities for development. Dissatisfied employees have a high propensity to engage in counterproductive behaviors and even leave the organization. If employees believe they are being treated and rewarded fairly, they are less likely to leave the organization. Both intrinsic satisfaction and extrinsic satisfaction are effective predictors of employees’ organizational commitment. In a sample of 214 Chinese employees at a state-owned steel company, job satisfaction and its facets had “a significant impact” on organizational commitment and its forms, in particular the affective component of commitment (Fu et al., 2011). Recent studies also suggest the predictive role of job satisfaction on organizational commitment (Ćulibrk et al., 2018; Lambert et al., 2020). Ćulibrk et al. (2018) investigated the relationship between job satisfaction and organizational commitment in a group of 566 participants working for eight companies in Serbia. They reported a moderate-to-strong correlation between the two factors.

As an important attitudinal variable, job satisfaction is also shown to be correlated with PsyCap. Li and colleagues investigated the relationships between western PsyCap and job satisfaction in a sample of 426 Chinese primary and middle school teachers (Li et al., 2011). The PsyCap of the teachers is significantly correlated with their job satisfaction. The teachers with higher PsyCap make more positive cognitive evaluations of work situations and are more able to seek and create positive conditions conducive to their development, which in turn leads to higher levels of job satisfaction. After the demographic variables being controlled for, PsyCap is found to be a direct predictor of job satisfaction. Still in the Chinese cultural setting, both task-oriented and guanxi-oriented PsyCaps are correlated with job satisfaction, with the latter correlation stronger than the former one (*ρ*=0.15 vs. 0.31; Ke and Sun, 2014). Based on these findings as well as the positive psychology theory, we believe that a high level of task-oriented and guanxi-oriented PsyCaps among the grassroots gongwuyuan cadres might enable them to cope with difficulties and stressors in their daily jobs, thus may help them feel more satisfied with their work, thereby reducing their intention of turnover and growing to be more committed to their organization. Consequently, we suggest as:

*H3a*: Job satisfaction will mediate the relation between task-oriented PsyCap and organizational commitment such that the relationship will be stronger when job satisfaction is high.

*H3b*: Job satisfaction will mediate the relation between guanxi-oriented PsyCap and organizational commitment such that the relationship will be stronger when job satisfaction is high.

Above the mediators of vocational identity and job satisfaction are proposed as the two crucial factors linking PsyCap to organizational commitment. While vocational identity is mainly concerned with an employee’s view toward their categorization and membership as a whole in a specific profession, job satisfaction focuses on individual experience as a worker in a given work environment. According to the social identity theory (Turner and Oakes, 1986), an individual’s social identity might help in understanding the group influence on the individual, such as their emotional state, motivation, and performance (Van Dick et al., 2006). Ulrich and colleagues suggested workgroup identification may influence employee job satisfaction during a major organizational change event that is often the result of large enterprise projects (Ullrich et al., 2005). Based on this theory and the above research on PsyCap and organizational commitment, it is hypothesized that as follows:

*H4a*: A chained mediation model could exist between task-oriented PsyCap and organizational commitment, with vocational identity and job satisfaction as two mediators.

*H4b*: A chained mediation model could exist between guanxi-oriented PsyCap and organizational commitment, with vocational identity and job satisfaction as two mediators.

**Figure 1:**
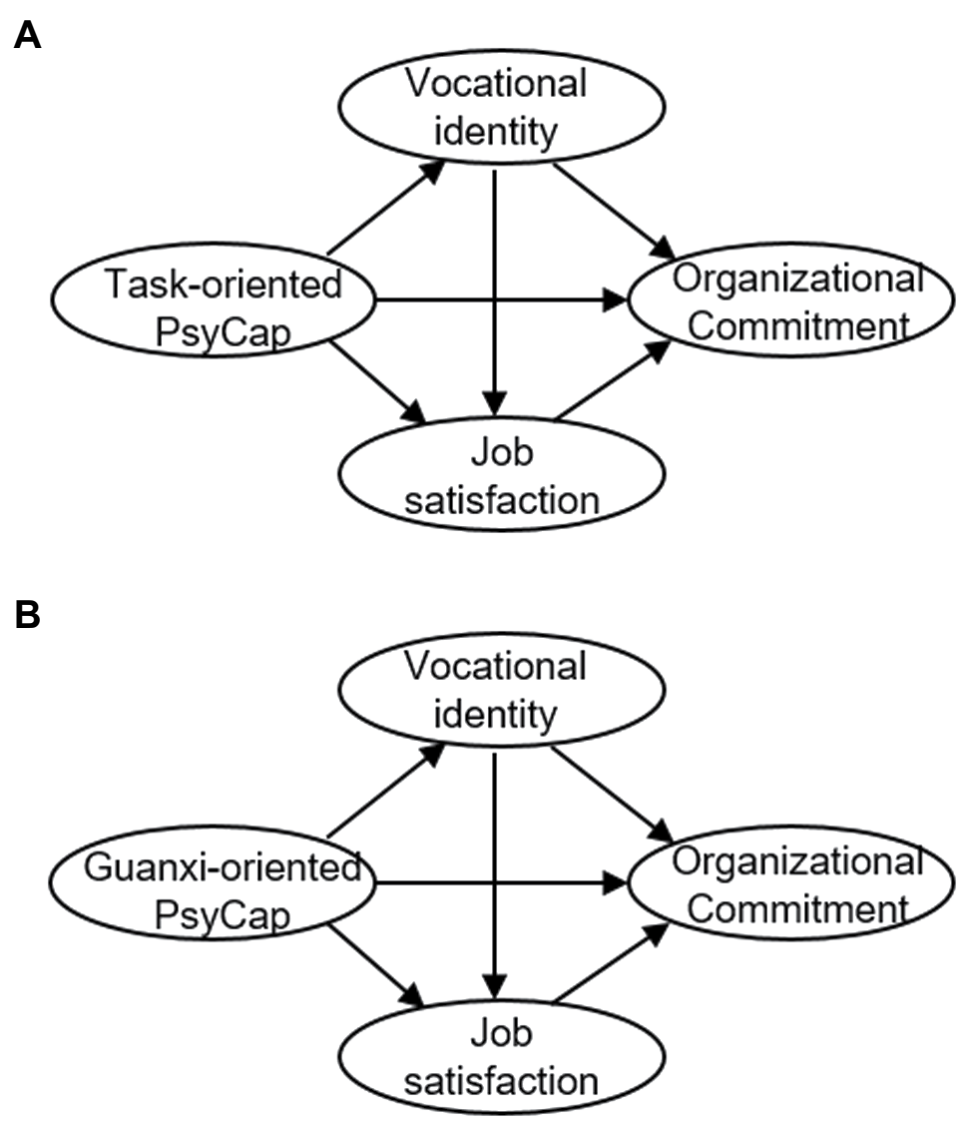
**(A)** The conceptual framework with task-oriented PsyCap. **(B)** The conceptual framework with guanxi-oriented PsyCap.

In summary, four sets of hypotheses are formulated based on previous studies and related theories. Based on these hypotheses, a conceptual framework on the PsyCap’s effects on organizational commitment was formed (Figures 1A,B). With this, we aim to examine the complex underlying mechanisms linking PsyCap to organizational commitment, through two potential chained mediators: vocational identity and job satisfaction. To address this aim, we collected data from a sample drawn from three townships in northwestern China. The ultimate goal is to shed light on the mechanisms of the grassroots gongwuyuan cadres’ behaviors and provide evidence for future prevention/intervention programs maintaining employees in government, especially in the rural areas.

**Table 1:** Means, standard deviations, and correlation matrix of variables.

Variables	*M*	*SD*	Task-oriented PsyCap	Guanxi-oriented PsyCap	Vocational identity	Job satisfaction
Task-oriented PsyCap	5.07	0.69
Guanxi-oriented PsyCap	5.18	0.61	0.82^**^
Vocational identity	3.97	0.50	0.57^**^	0.62^**^
Job satisfaction	4.08	0.48	0.72^**^	0.72^**^	0.67^**^
Organization commitment	3.76	0.49	0.70^**^	0.61^**^	0.48^**^	0.71^**^

** *p<0.01*.

## Materials and Methods

### Participants

Altogether a sample of 650 grassroots gongwuyuan cadres was selected in Gansu province, using a stratified sampling method. Three counties, Jinta, Pingchuan, and Qin’an were randomly chosen from the three representative cities corresponding to the west, middle, and east parts of Gansu. A total of 650 questionnaires were issued and 600 questionnaires were collected. Seventeen questionnaires were found invalid with missing data. So a total of 583 valid questionnaires was obtained for the final data analysis.

### Measures

#### Psychological Capital

We used the Chinese PsyCap Scale (Ke et al., 2009) to measure the task-oriented and guanxi-oriented PsyCaps. The Cronbach’s α coefficients for task-oriented PsyCap Scale, guanxi-oriented PsyCap Scale, and a composite scale consisting of the two PsyCaps were 0.85, 0.87, and 0.90, respectively. Task-oriented PsyCap and Guanxi-oriented PsyCap were of good criterion-related validity. Task-oriented PsyCap was related to task performance (0.43^**^), contextual performance (0.60^**^), job satisfaction (0.15^*^), job involvement (0.41^**^), and organizational commitment (0.21^**^). Guanxi-oriented PsyCap was related to task performance (0.42^**^), contextual performance (0.68^**^), traditionality (0.17^**^), interdependent self (0.52^**^), job satisfaction (0.31^**^), job involvement (0.45^**^), and organizational commitment (0.47^**^; Note: ^*^ *p*<0.01; ^**^: *p*<0.01). The Chinese PsyCap Scale also featured good construct validity. The confirmatory factor analysis showed *χ*^2^=1,469, df=731, *χ*^2^/df=2.01, GFI=0.85, CFI=0.90, RMSEA=0.070. In the current study, together 40 items were included to measure the four components of task-oriented PsyCap (enterprise and diligence, resiliency and perseverance, optimism and hope, and confidence and courage) and the four components of guanxi-oriented PsyCap (toleration and forgiveness, respect and courtesy, modesty and prudence, and thanksgiving and dedication). All responses were recorded on a 6-point Likert scale (1 = “strongly disagree” to 6 = “strongly agree”). The Cronbach’s α coefficient for the scale was 0.84.

#### Vocational Identity

We measured vocational identity with the 20-item Civil Servants Career Identity Scale (Liang, 2013). The scale included four factors: professional values, professional disposition, role values, and sense of belonging. A high vocational identity score on the scale means a stronger commitment to the job position. The coefficient alpha for this scale in the current study was 0.81.

#### Job Satisfaction

We measured job satisfaction with the short form of the minnesota satisfaction questionnaire (Weiss et al., 1967). The scale consists of 20 items, with 12 items measuring intrinsic satisfaction and 8 items measuring extrinsic satisfaction. The coefficient alpha for this scale in the current survey was 0.87.

#### Organizational Commitment

The organizational commitment measure comes from the Chinese Employees Organizational Commitment Scale (Ling et al., 2001). This scale, designed and developed specifically for the employees in China, reflects five dimensions of affective commitment, ideal commitment, economic commitment, normative commitment, and choice commitment. The participants were asked to indicate to the extent they agreed with the descriptions of commitments in a 7-point response scale, ranging from 1 (strongly disagree) to 7 (strongly agree). The test-retest reliability was above 0.8 for most of its items and the Cronbach’s α coefficient for this scale was 0.84.

### Data Collection and Processing

The grassroots gongwuyuan cadres in each township of the counties were contacted and informed about the survey beforehand. Voluntarily, they took part in a meeting where experimenters detailed the instructions as to how to finish questionnaires and answered their questions. All the participants gave consent forms before data were collected. All data were processed and analyzed using AMOS 23.0. The study was approved by the Research Ethics Committee of Lanzhou City University and carried out according to the regulations of the Committee as well as in line with the Helsinki Declaration.

## Results

### Common Method Deviation Analysis

Harman’s one-factor test was used to carry out the common method deviation analysis on all the valid data. The exploratory factor analysis (EFA) of all variables without rotation showed 22 factors featuring root values bigger than one. The variance of the first one was 29%, smaller than the critical value of 40%.

### Preliminary Analysis

Table1 displays the descriptive statistics and correlation results. Results revealed that both task-oriented PsyCap and guanxi-oriented PsyCap correlate positively with vocational identity, job satisfaction, and organizational commitment. The correlation coefficients are 0.57, 0.72, 0.70; 0.62, 0.72, and 0.61, respectively (*p*s<0.01), which showed that it was necessary to further reveal the internal relationship between these factors. The two types of PsyCap are also closely related (coefficient: 0.82, *p*<0.01).

### Multi-Collinearity Test

Multi-collinearity test was carried out (Table 2) to test any linearity existing between the two types of PsyCap, vocational identity and job satisfaction. All the four variance inflation factors (VIFs) are smaller than 5, suggesting the multi-collinearity between vocational identity, job satisfaction, task-oriented PsyCap, and Guanxi-oriented PsyCap was not an issue.

**Table 2:** Multi-collinearity test.

Independent variable	Tol	VIF
Vocational identity	0.51	1.95
Job satisfaction	0.37	2.74
Task-oriented PsyCap	0.29	3.48
Guanxi-oriented PsyCap	0.28	3.62

*Dependent variable: organizational commitment*.

### Measurement Model

**Figure 2:**
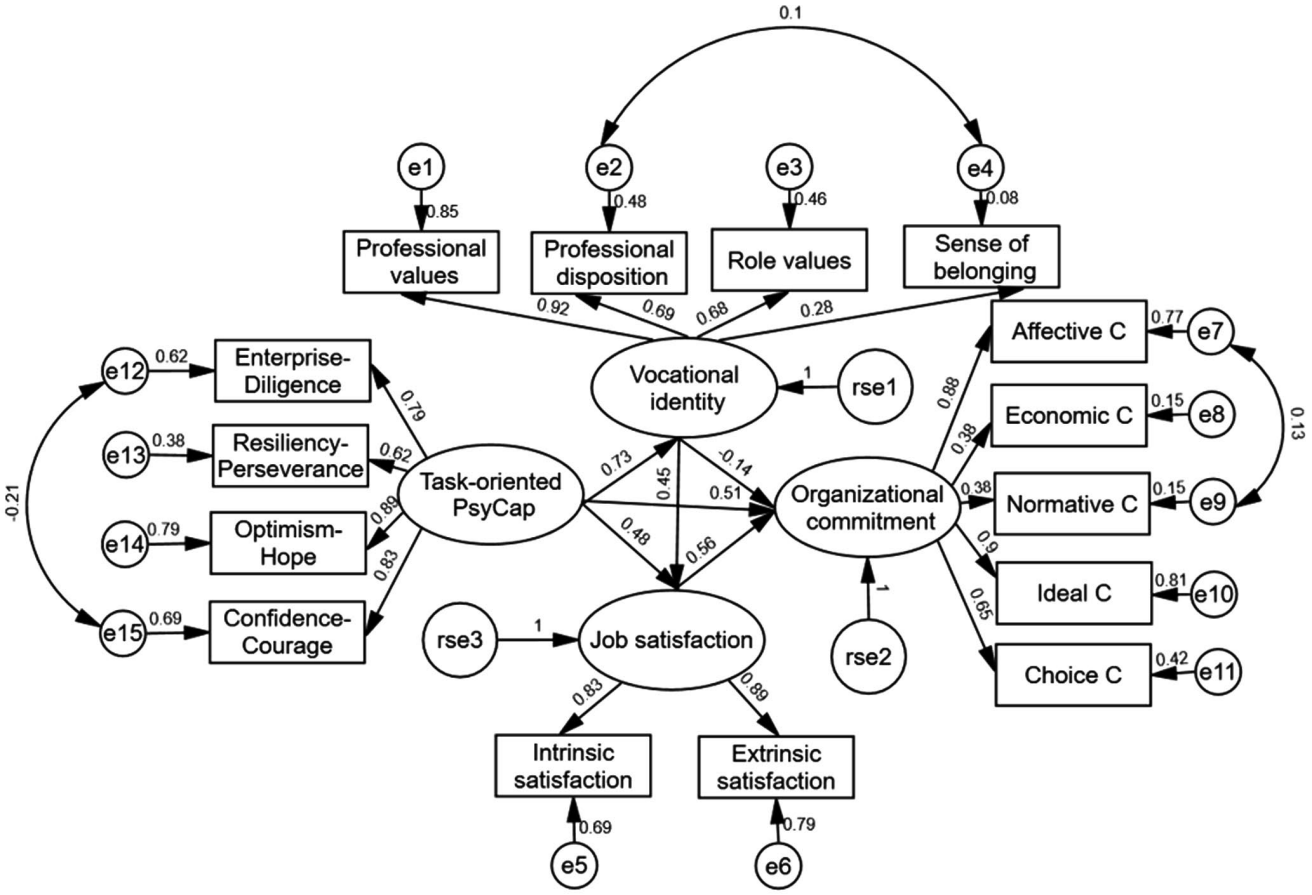
Chained mediation modeling of associations among task-oriented PsyCap, vocational identity, job satisfaction, and organizational commitment. C, commitment.

**Figure 3:**
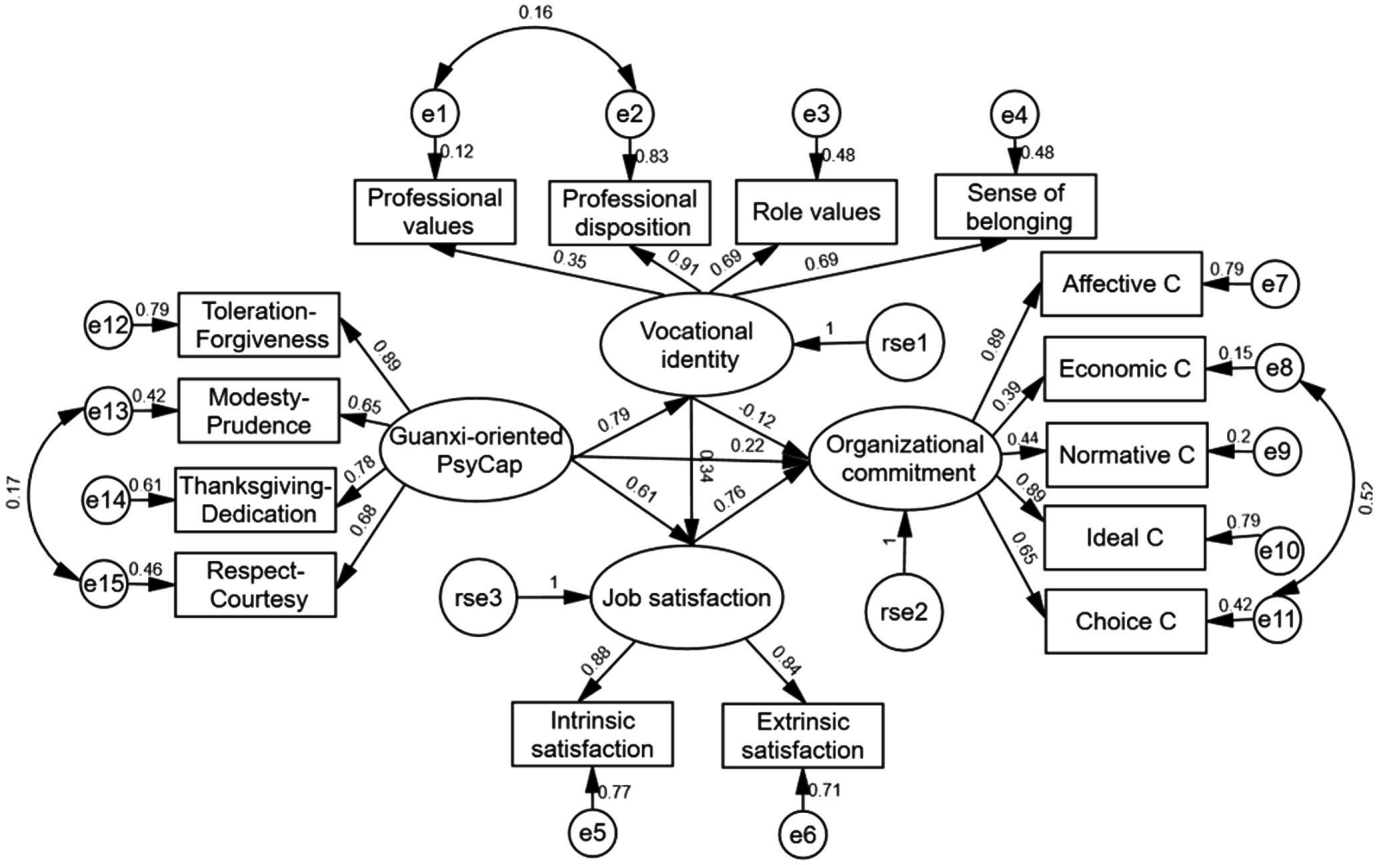
Chained mediation modeling of associations among guanxi-oriented PsyCap, vocational identity, job satisfaction, and organizational commitment. C, commitment.

The measurement models for task-oriented PsyCap and guanxi-oriented PsyCap are displayed in Figures 2, 3.

In Figure 2, the measurement model of task-oriented PsyCap included four latent factors of task-oriented PsyCap, Vocational identity, Organizational commitment, and Job satisfaction. Task-oriented PsyCap was composed of four explicit factors of Enterprise-Diligence (loading 0.79), Resiliency-Perseverance (0.62), Optimism-Hope (0.89), and Confidence-Courage (0.83). Their squared values were 0.62, 0.38, 0.79, and 0.69. Vocational identity was composed of four explicit variables of Professional values (0.92), Professional disposition (0.69), Role values (0.68), and Sense of belonging (0.28). Their squared values were 0.85, 0.48, 0.46, and 0.08. Job satisfaction was composed of two explicit factors of Intrinsic satisfaction (0.83) and Extrinsic satisfaction (0.89). Their squared values were 0.69 and 0.79. Organizational commitment was composed of five explicit factors of Affective commitment (0.88), Economic commitment (0.38), Normative commitment (0.38), Ideal commitment (0.90), and Choice commitment (0.65). Their squared values were 0.77, 0.15, 0.15, 0.81, and 0.42.

In Figure 3, the measurement model of guanxi-oriented PsyCap includes four latent factors of guanxi-oriented PsyCap, Vocational identity, Organizational commitment, and Job satisfaction. Guanxi-oriented PsyCap was composed of four explicit factors of Toleration-Forgiveness (0.89), Modesty-Prudence (0.65), Thanksgiving-Dedication (0.78), and Respect-Courtesy (0.68). Their variances explained by the latent variable were between 0.79, 0.42, 0.61, and 0.83. Vocational identity is composed of four explicit variables of Professional values (0.35), Professional disposition (0.91), Role values (0.69), and Sense of belonging (0.69). Their squared values were 0.12, 0.83, 0.48, and 0.48. Job satisfaction is composed of two explicit factors of Intrinsic satisfaction (0.88) and Extrinsic satisfaction (0.84). Their squared values were 0.77 and 0.71. Organizational commitment is composed of five explicit factors of Affective commitment (0.89), Economic commitment (0.39), Normative commitment (0.44), Ideal commitment (0.89), and Choice commitment (0.65). Their squared values were 0.79, 0.15, 0.20, 0.79, and 0.42.

### Structural Model: R^2^

In the structural model with task-oriented PsyCap as an exogenous latent variable, R^2^ of the three endogenous latent variables of vocational identity, job satisfaction, and organizational commitment were 0.54, 0.75, and 0.82, respectively. Task-oriented PsyCap explained 54% variance of vocational commitment; task-oriented PsyCap and vocational identity together explained 75% variance of job satisfaction; and task-oriented PsyCap, vocational identity, and job satisfaction together explained 82% variance of organizational commitment.

In the structural model with guanxi-oriented PsyCap as an exogenous latent variable, R^2^ of the three endogenous latent variables of vocational identity, job satisfaction, and organizational commitment were 0.62, 0.74, and 0.82, respectively. Task-oriented PsyCap explained 62% variance of vocational commitment; task-oriented PsyCap and vocational identity together explained 74% variance of job satisfaction; and task-oriented PsyCap, vocational identity, and job satisfaction together explained 82% variance of organizational commitment.

### From Task-Oriented PsyCap to Organizational Commitment: Modeling the Mediation Effects of Vocational Identity and Job Satisfaction

To control measurement errors, the current study used the mediating effect analysis procedure proposed by Wen and Ye (2014). The procedure, applicable to the observed variables and/or latent variables, is shown to be effective in terms of Type I error rate and power (Wen and Ye, 2014). The error-correction-based non-parametric percentage Bootstrap method was used to estimate confidence intervals of all coefficients. Task-oriented and guanxi-oriented PsyCaps, vocational identity, job satisfaction, and organizational commitment were treated as latent variables. The factors of each of these variables were treated as explicit indicator variables.

The goodness-of-fit of the models was evaluated using absolute and relative indices. The absolute goodness-of-fit indices calculated are the chi-square goodness-of-fit statistic, RMSEA, GFI, NNFI, IFI, and CFI. Non-significant values of chi-square indicated that the hypothesized model fitted the data. The values of RMSEA between 0.05 and 0.08 indicated an acceptable fit while a value greater than 0.1 means the model should be rejected. Relative-fit index values greater than 0.90 suggested a good fit.

We first tested the effect of task-oriented PsyCap on organizational commitment, i.e., testing the significance of C. The findings showed that the model fits well, with RMSEA=0.08, SRMR=0.04, CFI=0.98, and NNFI=0.96. The PsyCap was found to be a significant predictor of organizational commitment (*β*=0.88, *p*<0.001). Therefore, Hypothesis 1a was supported. Then, we continued to add vocational identity and job satisfaction as mediating variables in the model. The result shows that the model fitted well, RMSEA=0.08, SRMR=0.05, CFI=0.98, and NNFI=0.96. The predictive effect of the PsyCap on vocational identity reached significance (*β*=0.73, *p*<0.001). Vocational identity was a significant predictor of job satisfaction (*β*=0.45, p<0.001), and job satisfaction was a significant predictor of organizational commitment (*β*=0.56, *p*<0.001). This showed that the higher the PsyCap ratings of gongwuyuan cadres, the stronger their vocational identity could be. Strong identification with their profession will in turn increase their job satisfaction and ultimately their commitment to their organizations. See Figure 2 for the modeling.

The Bias-Corrected Percentile Bootstrap (BCPB) method (using 3,000 random bootstrap samples) was used to test the mediating effect. This method was used over alternative tests (e.g., the Sobel test) as it allows us to avoid Type I errors that may result from non-normal distributions of an indirect effect as well as it accommodates small- and medium-sized samples (Mackinnon et al., 2004). As shown in Table 3, the mediating effect of vocational identity between task-oriented PsyCap and organizational commitment was not significant, 95% CI [−0.01, 0.01]. Therefore, Hypothesis 2a was not supported. The mediating effect of job satisfaction between PsyCap and organizational commitment was 0.27, 95% CI [0.03, 0.20]. Thus, Hypothesis 3a was confirmed. The chained mediating effect of vocational identity and job satisfaction was also significant (0.18), 95% CI [0.01, 0.28]. Therefore, Hypothesis 4a was supported. All things considered vocational identity and job satisfaction played a chained mediating effect between PsyCap and organizational commitment.

**Table 3:** Testing the mediation effects of vocational identity and job satisfaction in Model 1.

Path	Mediating effect size	Confidence interval
Task-oriented PsyCap—Vocational identity—Organizational commitment	0	[−0.01, 0.01]
Task-oriented PsyCap—Job satisfaction—Organizational commitment	0.27	[0.03, 0.20]
Task-oriented PsyCap—Vocational identity—Job satisfaction—Organizational commitment	0.18	[0.01, 0.28]

### From Guanxi-Oriented PsyCap to Organizational Commitment: Modeling the Mediation Effects of Vocational Identity and Job Satisfaction

The same method as the above was used in testing the model involving guanxi-oriented PsyCap. With the tested significance of C, the result showed a good fit in modeling guanxi-oriented PsyCap and other consequence variables, RMSEA=0.07, SRMR=0.05, CFI=0.98, and NNFI=0.95. The guanxi-oriented PsyCap was a significant predictor of organizational commitment (*β*=0.81, *p*<0.001). Therefore, Hypothesis 1b was validated. This model remained fit with the addition of vocational identity and job satisfaction as mediators, RMSEA=0.07, SRMR=0.05, CFI=0.96, and NNFI=0.94. As shown in Figure 3, the guanxi-oriented PsyCap was a positive predicator of vocational identity (*β*=0.79, *p*<0.001), vocational identity a positive predictor of job satisfaction (*β*=0.34, p<0.001), and job satisfaction a positive predictor of organizational commitment (*β*=0.76, *p*<0.001).

The same BCPB method as above was used in the mediating effect test. As shown in Table 4, the mediating effect of vocational identity between guanxi-oriented PsyCap and organizational commitment was not significant, 95% CI [−0.25, 0.07]. Therefore, Hypothesis 2b was not supported. The mediating effect of job satisfaction between guanxi-oriented PsyCap and organizational commitment was 0.47, 95% CI [0.17, 0.89]. Thus, Hypothesis 3b was supported. The chained mediating effect of vocational identity and job satisfaction reached significance (0.20), 95% CI [0.04, 0.51]. Therefore, Hypothesis 4b was supported. Taken together, vocational identity and job satisfaction chain-mediated guanxi-oriented PsyCap and organizational commitment.

**Table 4:** Testing the mediation effects of vocational identity and job satisfaction in Model 2.

Path	Mediating effect size	Confidence interval
Guanxi-oriented PsyCap—Vocational identity—Organizational commitment	0	[−0.25, 0.07]
Guanxi-oriented PsyCap—Job satisfaction—Organizational commitment	0.47	[0.17, 0.89]
Guanxi-oriented PsyCap—Vocational identity—Job satisfaction—Organizational commitment	0.20	[0.04, 0.51]

## Discussion

Although PsyCap and its consequence variables have remained a hot subject of inquiry for decades, their role in maintaining a sustainable workforce in underdeveloped rural areas has been largely unknown. Still unknown are the effects of positive resources in the collectivist culture setting which is, to some extent, different from those in the western culture where PsyCap has been researched mostly. The current study is among the few that addressed these concerns. It explored the complex relationships among PsyCap, vocational identity, job satisfaction, and organizational commitment in a sample of grassroots gongwuyuan cadres in the rural areas of northwest Gansu province of China. Both task-oriented and guanxi-oriented PsyCaps positively predict organizational commitment. Vocational identity and job satisfaction work as mediators in the chained relationship between task-oriented/guanxi-oriented PsyCap and organizational commitment. Vocational identity alone does not fulfill the role of a mediator between task-oriented/guanxi-oriented PsyCap and organizational commitment. In the following, these findings will be discussed in detail.

### The Effect of PsyCap on Organizational Commitment

Task-oriented and guanxi-oriented PsyCaps were found to be positive predictors of organizational commitment. This is consistent with previous studies in China (Ke and Sun, 2014; Xu et al., 2020) and with the findings in the west (Larson and Luthans, 2006; Newman et al., 2018). Thus, our study provides further support for the association between PsyCap and organizational commitment. In the current study, the predictive effect of task-oriented and guanxi-oriented PsyCaps on organizational commitment of gongwuyuan cadres reached 0.88 and 0.81, respectively, suggesting a major role of the two types of PsyCap. Those cadres characterized by high task-oriented PsyCap states, such as optimism and resiliency, are likely to make progress and improve their job performance and remain committed to the local governments they work for. PsyCap goes beyond human capital (e.g., knowledge, skills, perspectives, and abilities) and social capital (e.g., trust and relationships) and focuses on who you are (e.g., self-confidence, hope, optimism, and resilience; Luthans et al., 2005). The four sets of positive PsyCap among the gongwuyuan cadres, namely, confidence-courage, optimism-hope, resiliency-perseverance, and enterprise-diligence, may be closely related to their values, ideals, and personal interests and traits. The later factors as well as wages and benefits of work influence their organizational commitment, which measures an emotional attachment to the organization (Zhong, 2007). Notably, in comparison with the western PsyCap scale which included hope, optimism, and resiliency (Luthans et al., 2005), the local task-oriented PsyCap scale featured an addition of two factors of enterprise-diligence and confidence-courage (Ke et al., 2009), which were emergent through POB criterion-meeting standard and careful data collection and analysis procedures (detailed in Introduction). According to Ke et al. (2009; in Table 2), the eigenvalues of enterprise-diligence, resiliency-perseverance, optimism-hope, and confidence-courage were 7.61, 2.94, 1.68, and 1.34, which explained 28.18, 10.88, 6.20, and 4.97% variance of the task-oriented PsyCap scale. In the EFA of the task-oriented PsyCap Scale, each factorial loading of the four sets of enterprise-diligence, resiliency-perseverance, optimism-hope, and confidence-courage was larger than 0.60. The four sets together explained 54.68% of the total variance. Each Cronbach’s α coefficient of the four elements was higher than 0.70. These additional elements of the task-oriented PsyCap were also replicated in different samples in China, such as primary and middle school teachers (Wu et al., 2012), doctors (Sun et al., 2014), employees in a company and a local government in Beijing (Ke and Sun, 2014), and those working in the aviation industry (Wang, 2020). In comparison with the western version of PsyCap (Luthans et al., 2005), the task-oriented PsyCap was found to explain more variances of employee’s task performance [(*β*=0.453 vs. 0.421); Ke et al., 2009]. In a meta-analysis, it is found that task performance and organizational commitment are significantly correlated (Wang et al., 2019). So the additional enterprise-diligence and confidence-courage might also contribute to the relatively high predictive power of our task-oriented PsyCap in the Chinese cultural setting. Guanxi-oriented PsyCap represents the qualities of modesty, respect, toleration, and devotion which arguably are facilitators in communications between colleagues in an organization. In China, both harmonious relationships between individuals and between organizations are regarded as highly important in their achievements. The strong predictive power of the guanxi-oriented PsyCap, therefore, bolsters this cultural characteristic. It also highlights the importance of these qualities in grassroots cadres in fostering their commitment to their organizations and reducing job turnover.

While the PsyCaps were shown to be strong predictors of organizational commitment, no evidence of the mediating effect of vocational identity was found. This result suggests that vocational identity alone cannot serve as a mediator connecting task-oriented/guanxi-oriented PsyCap and organizational commitment. Previous studies do show that components of task-oriented PsyCap are statistically significant predictors of vocational identity (Wang et al., 2013), which also impacts on turnover intention, an outcome variable of organizational commitment (Wang et al., 2020). Meanwhile, some of the attributes of guanxi-oriented PsyCap are found to benefit social contacts in the collectivist cultures (Vuyk et al., 2020), which presumably influences vocational identity. So the null mediating effect of vocational identity of the grassroots cadres might be due to an absence of an accompanying variable in associating PsyCaps and organizational commitment. Therefore, it is necessary to further explore the complex dynamics underlying the relation between the PsyCaps and organizational commitment, with job satisfaction and vocational identity as two possible critical factors involved.

### The Mediating Role of Job Satisfaction Between PsyCap and Organizational Commitment

In our study, job satisfaction serves as a mediator between PsyCap and organizational commitment. It suggests an indirect effect of PsyCap on organizational commitment, through the mediating effect of job satisfaction. This result is consistent with previous findings documenting the positive effects of PsyCap (Schulz et al., 2014; Karakus et al., 2019). Karakus et al. (2019) investigated the effects of PsyCap on organizational commitment in a sample of primary school teachers in Turkey. They reported that PsyCap functions as a predictor of the organizational commitment of teachers (as well as motivation and intent to leave), through the mediator of job satisfaction. According to the Duality Theory of Job Satisfaction (Herzberg et al., 1959), the work dimensions are classified into motivators and hygiene factors. Motivators refer to the six satisfying events of achievement, recognition, responsibility, advancement, work itself, and growth. Hygiene factors are mainly disruptions in the external work, such as supervision, salary, administration, personal life, and relationships with subordinates. Job satisfaction can be improved only by the motivator factors which are concerned about the internal states of mind. The positive psychological qualities of the gongwuyuan cadres in the rural governments, such as confidence, courage, hope, and optimism, will equip them with the energy, initiative, and self-discipline to better work performance. All these contribute to high job satisfaction. Employees with high (task-oriented) PsyCap are found to possess the capacity of self-regulation that provides the energy, initiative, and discipline necessary to accomplish goals (Bandura, 1997; Luthans and Youssef, 2007). In a sample of 231 employees in different companies in Pakistan, Abbas et al. (2014) found that PsyCap leads to more confidence and more positive thinking in employees and importantly results in better work performance and higher job satisfaction. Likewise, the guanxi-oriented PsyCap was also found to correlate with job satisfaction. This is in agreement with the previous finding in China (Ke and Sun, 2014). The resulted satisfaction with their job from their task-oriented and guanxi-oriented PsyCaps can be internalized as their positive attitude to their organization. This may help them to become attached to the organization and committed to their work.

### Vocational Identity and Job Satisfaction Work as Mediators Between Task-Oriented and Guanxi-Oriented PsyCaps and Organizational Commitment

Vocational identity and job satisfaction are closely related. Both are mediators in the chained relation between PsyCap and organizational commitment. Though no evidence of vocational identity as a mediator was found, the variable plays its role through job satisfaction, thus building the chained mediators linking PsyCap and organizational commitment. With an inherent need for identification with their organization (Van Dick et al., 2005), the gongwuyuan cadres’ thoughts, feelings, and even actions might be positively influenced. In China, gongwuyuan cadres enjoy a relatively high and stable social position and prestige (Smith, 2015). Still, the positive personal attributes, such as courage and motivation, may help the cadres fit in with their work environment and in turn, enhance their vocational identity. Such an identification plus positive internal characteristics may well result in betterment in their perception toward their daily jobs and become increasingly committed to their organizations. While previous studies did not adequately address the link between PsyCap and organizational commitment (Miao et al., 2013; Sun and Ye, 2015), the current study is among the few that explores this area. It clarifies the underlying mechanisms associating PsyCap with employees’ attitudinal variables. The positive influence of PsyCaps on organizational commitment is realized through the chained mediators of two attitudinal variables: vocational identity and job satisfaction. It supports the critical role of PsyCap in shaping employees’ job performance and organizational commitment (Van Dick et al., 2005; Karakus et al., 2019). It also lends support to social identity theory in that the positive task-oriented and guanxi-oriented psychological capitals of members in an organization themselves can influence their identification (Turner and Oakes, 1986). Positive psychological capitals can benefit them in forming a strong identification with their organization. This might be attributed to the collectivist culture in China where employees value team spirit and develop a sense of belonging toward the organization they work for and honor them (Smith, 2015). This is true especially in the state-owned enterprises and government agencies which feature stability and relatively good work-family balance and logistics support. The resulted satisfaction toward their careers leads to their commitment to the government agencies they work for.

### Implications

Our findings have several practical implications. First of all, boosting gongwuyuan cadres’ positive task-oriented and guanxi-oriented PsyCaps are critical in maintaining a steady and committed pool of workforce. As can be seen from our chained mediating analysis, the two PsyCaps initiate a chained mediation linking them to organizational commitment. What supervisors and managers need to do first is to demonstrate positive leadership through PsyCap development. Being “more positive, authentic, transparent, and trustworthy” leaders enable them to be role models for their employees and help create necessary resources and the positive climate in an organization necessary for them to develop their PsyCaps (Luthans and Youssef-Morgan, 2017). In recruiting employees, in addition to their performance in written exams, such as the Annual Gongwuyuan Examination in China, their psychological attributes need to be taken into consideration. Those with high task-oriented PsyCap states, such as motivation, resiliency, and perseverance, and guanxi-oriented PsyCap states, such as toleration, respect, modesty, and thanksgiving, should be valued in the selection process. Still, various intervention methods and programs may be used to enhance the PsyCap levels of existing employees, such as the psychological capital intervention model and gamification (Luthans and Youssef-Morgan, 2017), though cross-cultural adaptations are needed. Secondly, the chained mediators of vocational identity and job satisfaction suggest their roles in promoting employees’ commitment to their organization. Managers should train and supervise employees to build up their work identification by focusing on the positive image of public service in government, creating role models, promoting a harmonious work climate, and practicing healthy inter-colleague and employer-employee interactions. All these measures are key to cultivating and developing civil servants’ commitment to their work and adherence to the professional norms.

### Limitations

The current study is not without limitations. First, with a cross-sectional research design, this study cannot warrant a causal relationship linking PsyCap and organizational commitment. Researchers may endeavor to conduct experimental studies to test the potential causal impact of PsyCap training intervention on organizational commitment. Second, the sampled data were collected in one of the most underprivileged townships in Gansu, a less developed province in western China. Caution may be applied in attempts to generalize the findings to other sectors or to those relatively well-off areas. Third, with questionnaires, we are not 100 percent sure our respondents gave the accurate answers they intended to provide. Further studies may overcome this by utilizing a mixed-method approach.

## Conclusion

Notwithstanding the above limitations, our study is among the few to provide a chained mediation analysis of the link of the variables of PsyCap, vocational identity, job satisfaction, and organizational commitment in underprivileged rural areas. Taking into account the cultural differences in psychological resources, the current study measured the local PsyCap in China, which is composed of task-oriented PsyCap and guanxi-oriented PsyCap. The task-oriented PsyCap is similar to the western PsyCap while guanxi-oriented PsyCap is unique in the Chinese context. Based on a sample of gongwuyuan cadres at the grassroots level of government in northwestern China, it is found that task-oriented and guanxi-oriented PsyCaps are significant predictors of organizational commitment. They influence organizational commitment through vocational identity and job satisfaction, which are two mediators in the chained mechanism among the four variables. Given the gongwuyuan cadres as part of the new employee workforce in today’s China featuring more individualistic perspectives into their career development, it is crucial that employers manage and develop their PsyCap in order to maintain a sustainable workforce, especially in the rural areas.

## Data Availability Statement

The raw data supporting the conclusions of this article will be made available by the authors, without undue reservation.

## Ethics Statement

The studies involving human participants were reviewed and approved by the Research Ethics Committee of Lanzhou City University. The patients/participants provided their written informed consent to participate in this study.

## Author Contributions

CX, DW, and JZ: Conceptualization and writing—review and editing. CX and JL: methodology. CX, JZ, and JL: software and formal analysis. CX: investigation. CX and DW: writing—original draft preparation, visualization, and funding acquisition. DW, JL, and JZ: supervision. project administration, CX: project administration. All authors have read and agreed to the published version of the manuscript.

## Conflict of Interest

The authors declare that the research was conducted in the absence of any commercial or financial relationships that could be construed as a potential conflict of interest.

## Publisher’s Note

All claims expressed in this article are solely those of the authors and do not necessarily represent those of their affiliated organizations, or those of the publisher, the editors and the reviewers. Any product that may be evaluated in this article, or claim that may be made by its manufacturer, is not guaranteed or endorsed by the publisher.

## Footnotes

^1^Under the supervision of the central government, the local government in China is composed of four different levels in a hierarchy, the township, county, prefecture or municipality, and the province. Each level in the structure is responsible for supervising the work done by lower levels on the administrative strata. Under the county level, the township, where villages (usually around a 100 families per village) are governed, is normally considered the grassroots in rural areas. Starting from the 1990s, the national system of civil servants (gongwuyuan in Mandarin Chinese) was launched and has been undergoing constant reform ever since. Currently, all the regular staff working in the grassroots government and its various branch offices are roughly categorized as gongwuyuan cadres, spanning levels 10–15 in the national gongwuyuan scale. Participants in the current study are the gongwuyuan cadres of various levels in the local administration system of the townships.
